# Care of patients with inborn errors of immunity in thirty J Project countries between 2004 and 2021

**DOI:** 10.3389/fimmu.2022.1032358

**Published:** 2022-12-16

**Authors:** Hassan Abolhassani, Tadej Avcin, Nerin Bahceciler, Dmitry Balashov, Zsuzsanna Bata, Mihaela Bataneant, Mikhail Belevtsev, Ewa Bernatowska, Judit Bidló, Péter Blazsó, Bertrand Boisson, Mikhail Bolkov, Anastasia Bondarenko, Oksana Boyarchuk, Anna Bundschu, Jean-Laurent Casanova, Liudmyla Chernishova, Peter Ciznar, Ildikó Csürke, Melinda Erdős, Henriette Farkas, Daria S. Fomina, Nermeen Galal, Vera Goda, Sukru Nail Guner, Péter Hauser, Natalya I. Ilyina, Teona Iremadze, Sevan Iritsyan, Vlora Ismaili-Jaha, Milos Jesenak, Jadranka Kelecic, Sevgi Keles, Gerhard Kindle, Irina V. Kondratenko, Larysa Kostyuchenko, Elena Kovzel, Gergely Kriván, Georgina Kuli-Lito, Gábor Kumánovics, Natalja Kurjane, Elena A. Latysheva, Tatiana V. Latysheva, István Lázár, Gasper Markelj, Maja Markovic, László Maródi, Vafa Mammadova, Márta Medvecz, Noémi Miltner, Kristina Mironska, Fred Modell, Vicki Modell, Bernadett Mosdósi, Anna A. Mukhina, Marianna Murdjeva, Györgyi Műzes, Umida Nabieva, Gulnara Nasrullayeva, Elissaveta Naumova, Kálmán Nagy, Beáta Onozó, Bubusaira Orozbekova, Malgorzata Pac, Karaman Pagava, Alexander N. Pampura, Srdjan Pasic, Mery Petrosyan, Gordana Petrovic, Lidija Pocek, Andrei P. Prodeus, Ismail Reisli, Krista Ress, Nima Rezaei, Yulia A. Rodina, Alexander G. Rumyantsev, Svetlana Sciuca, Anna Sediva, Margit Serban, Svetlana Sharapova, Anna Shcherbina, Brigita Sitkauskiene, Irina Snimshchikova, Shqipe Spahiu-Konjusha, Miklós Szolnoky, Gabriella Szűcs, Natasa Toplak, Beáta Tóth, Galina Tsyvkina, Irina Tuzankina, Elena Vlasova, Alla Volokha

**Affiliations:** ^1^Research Center for Immunodeficiencies, Pediatrics Center of Excellence, Children’s Medical Center, Tehran University of Medical Sciences, Tehran, Iran; ^2^Children’s Hospital, University Medical Center Ljubljana, Ljubljana, Slovenia; ^3^Division of Pediatric Allergy and Immunology, Near East University, Nicosia, Cyprus; ^4^Department of Hematopoietic Stem Cell Transplantation, Dmitry Rogachev National Medical Center of Pediatric Hematology, Oncology and Immunology, Moscow, Russia; ^5^Department of Dermatology and Allergology, University of Szeged, Szeged, Hungary; ^6^Department of Immunology, Clinical Emergency Paediatric Hospital Louis Turcanu, Timisoara, Romania; ^7^Immunology Department, Belarussian Research Center for Pediatric Oncology, Hematology and Immunology, Minsk, Belarus; ^8^Department of Immunology, The Children’s Memorial Health Institute, Warsaw, Poland; ^9^National Health Insurance Fund Administration, Budapest, Hungary; ^10^Department of Pediatrics, University of Szeged, Szeged, Hungary; ^11^St. Giles Laboratory of Human Genetics of Infectious Diseases, Rockefeller Branch, The Rockefeller University, New York, NY, United States; ^12^Laboratory of Human Genetics of Infectious Diseases, Necker Branch, INSERM, Necker Hospital for Sick Children, Paris, France; ^13^Paris Cité University, Imagine Institute, Paris, France; ^14^Department of Pediatrics, Necker Hospital for Sick Children, Paris, France; ^15^Howard Hughes Medical Institute, New York, NY, United States; ^16^Institute of Immunology and Physiology of the Ural Branch of the Russian Academy of Sciences, Yekaterinburg, Russia; ^17^Pediatric Infectious Disease and Pediatric Immunology Department, Shupyk National Healthcare University of Ukraine, Kyiv, Ukraine; ^18^Department of Children’s Diseases and Pediatric Surgery, I.Horbachevsky Ternopil National Medical University, Ternopil, Ukraine; ^19^Department of Pediatrics, University of Bratislava, Bratislava, Slovakia; ^20^Department of Pediatrics, Jósa András County Hospital and University Teaching Hospital, Nyíregyháza, Hungary; ^21^Primary Immunodeficiency Clinical Unit and Laboratory, Department of Dermatology, Venereology and Dermatooncology, Semmelweis University, Budapest, Hungary; ^22^Center for Hereditary Angioedema, Department of Internal Medicine and Hematology, Semmelweis University, Budapest, Hungary; ^23^Department of Clinical Immunology, Sechenov First Moscow State Medical University, Moscow, Russia; ^24^Pediatrics Department, Faculty of Medicine, Cairo University, Giza, Egypt; ^25^Department of Pediatric Hematology and Stem Cell Transplantation, Central Hospital of Southern Pest, Budapest, Hungary; ^26^Department of Pediatric Immunology, Necmettin Erbakan University, Konya, Turkey; ^27^Velkey László Child’s Health Center, Borsod-Abaúj-Zemplén County Hospital and University Teaching Hospital, Miskolc, Hungary; ^28^Department of Pulmonology, National Research Center Institute of Immunology, Federal Biomedical Agency of Russia, Moscow, Russia; ^29^Department of Pulmonology, Iashvili Children’s Central Hospital, Tbilisi, Georgia; ^30^Department of Hematology and Transfusion Medicine, National Institute of Health, Yerevan, Armenia; ^31^Pediatric Clinic, Department of Gastroenterology, University Clinical Center of Kosovo Faculty of Medicine, University of Prishtina “Hasan Prishtina”, Pristina, Kosovo; ^32^Department of Pediatrics, Jessenius Faculty of Medicine in Martin, Comenius University in Bratislava, University Hospital in Martin, Martin, Slovakia; ^33^Department of Pediatrics, Division of Clinical Immunology, Allergology, Respiratory Diseases and Rheumatology, University Hospital Center Zagreb, Zagreb, Croatia; ^34^Institute for Immunodeficiency, Medical Center - University of Freiburg, Faculty of Medicine, University of Freiburg, Freiburg, Germany; ^35^Russian Children’s Clinical Hospital of the N.I. Pirogov Russian National Research Medical University, Ministry of Health of Russia, Moscow, Russia; ^36^Department of Pediatric Immunology and Reumatology, Western-Ukrainian Specialized Children’s Medical Centre, Lviv, Ukraine; ^37^Program of Clinical Immunology, Allergology and Pulmonology, University Medical Center, Nazarbaev University, Nur-Sultan, Kazakhstan; ^38^Department of Pediatrics, University Hospital Centre Mother Theresa, Tirana, Albania; ^39^Department of Rheumatology and Immunology, Faculty of Medicine, University of Pécs, Pécs, Hungary; ^40^Department of Biology and Microbiology, Rīga Stradiņš University, Riga, Latvia; ^41^Department of Meteorology, University of Debrecen, Debrecen, Hungary; ^42^Department of Eastern Europe, Octapharma Nordic, Stockholm, Sweden; ^43^Research-Immunology Laboratory, Azerbaijan Medical University, Baku, Azerbaijan; ^44^Department of Biochemistry and Molecular Biology, Faculty of Medicine, University of Debrecen, Debrecen, Hungary; ^45^University Clinic for Children’s Diseases, Department of Immunology, Faculty of Medicine, University “St.Cyril and Methodius”, Skopje, North Macedonia; ^46^The Jeffrey Modell Foundation, New York, NY, United States; ^47^Department of Pediatrics, Clinical Center, University of Pécs, Pécs, Hungary; ^48^Department of Microbiology and Immunology, Faculty of Pharmacy, Research Institute, Medical University-Plovdiv, Plovdiv, Bulgaria; ^49^Department of Internal Medicine and Hematology, Semmelweis University, Budapest, Hungary; ^50^Institute of Immunology and Human Genomics, Academy of Sciences of the Republic of Uzbekistan, Tashkent, Uzbekistan; ^51^1-st Pediatric Department, Azerbaijan Medical University, Baku, Azerbaijan; ^52^Department of Clinical Immunology, Faculty of Medicine, Alexandrovska Hospital, Medical University, Sofia, Bulgaria; ^53^Department of Epidemiology and Immunology, Kyrgyz-Russian Slavic University, Bishkek, Kyrgyzstan; ^54^Department of Child and Adolescent Medicine, Tbilisi State Medical University, Tbilisi, Georgia; ^55^Department of Allergology and Clinical Immunology, Veltischev Research and Clinical Institute for Pediatrics and Pediatric Surgery of the Pirogov Russian National Research Medical University of the Russian Ministry of Health, Moscow, Russia; ^56^Department of Pediatric Immunology, Mother and Child Health Institute, Belgrade, Serbia; ^57^Department of Hematology and Transfusion Medicine, Pediatric Cancer and Blood Disorders Center, Yerevan, Armenia; ^58^Department of Allergology, Institute for Children Diseases, Clinical Center of Montenegro, Podgorica, Montenegro; ^59^Department of Pediatrics, Speransky Children’s Municipal Clinical Hospital #9, Moscow, Russia; ^60^Department of Pediatrics, Center of Allergology and Immunology, East-Tallinn Central Hospital, Tallinn, Estonia; ^61^Research Center for Immunodeficiencies, Children’s Medical Center, Tehran University of Medical Sciences, Tehran, Iran; ^62^Department of Pulmonology, Nicolae Testemitanu State University of Medicine and Pharmacy, Chisinau, Moldova; ^63^Department of Pulmonology, Motol University Hospital, 2nd Faculty of Medicine, Charles University, Prague, Czechia; ^64^Academy of Medical Sciences-Research Unit, Clinical Emergency Paediatric Hospital Louis Turcanu, Timisoara, Romania; ^65^Department of Immunology and Allergology, Lithuanian University of Health Sciences, Kaunas, Lithuania; ^66^Medical Institute, Orel State University named after I.S.Turgenev, Orel, Russia; ^67^Pediatric Clinic, Genetics Department, University Clinical Center of Kosovo Faculty of Medicine, University of Pristina ”Hasan Prishtina”, Pristina, Kosovo; ^68^Primary Immunodeficiency Clinic, Szent János Hospital, Budapest, Hungary; ^69^Department of Rheumatology, Faculty of Medicine, University of Debrecen, Debrecen, Hungary; ^70^Institute of Laboratory Medicine, Faculty of Medicine, University of Debrecen, Debrecen, Hungary; ^71^Department of Territorial Clinical Center of Specialized Types of Medical Care, State Autonomous Health Care Institution, Vladivostok, Russia

**Keywords:** J Project, immunodeficiencies, Eastern and Central Europe, Asia, ESID, parameters

## Abstract

**Introduction:**

The J Project (JP) physician education and clinical research collaboration program was started in 2004 and includes by now 32 countries mostly in Eastern and Central Europe (ECE). Until the end of 2021, 344 inborn errors of immunity (IEI)-focused meetings were organized by the JP to raise awareness and facilitate the diagnosis and treatment of patients with IEI.

**Results:**

In this study, meeting profiles and major diagnostic and treatment parameters were studied. JP center leaders reported patients’ data from 30 countries representing a total population of 506 567 565. Two countries reported patients from JP centers (Konya, Turkey and Cairo University, Egypt). Diagnostic criteria were based on the 2020 update of classification by the IUIS Expert Committee on IEI. The number of JP meetings increased from 6 per year in 2004 and 2005 to 44 and 63 in 2020 and 2021, respectively. The cumulative number of meetings per country varied from 1 to 59 in various countries reflecting partly but not entirely the population of the respective countries. Altogether, 24,879 patients were reported giving an average prevalence of 4.9. Most of the patients had predominantly antibody deficiency (46,32%) followed by patients with combined immunodeficiencies (14.3%). The percentages of patients with bone marrow failure and phenocopies of IEI were less than 1 each. The number of patients was remarkably higher that those reported to the ESID Registry in 13 countries. Immunoglobulin (IgG) substitution was provided to 7,572 patients (5,693 intravenously) and 1,480 patients received hematopoietic stem cell therapy (HSCT). Searching for basic diagnostic parameters revealed the availability of immunochemistry and flow cytometry in 27 and 28 countries, respectively, and targeted gene sequencing and new generation sequencing was available in 21 and 18 countries. The number of IEI centers and experts in the field were 260 and 690, respectively. We found high correlation between the number of IEI centers and patients treated with intravenous IgG (IVIG) (correlation coefficient, cc, 0,916) and with those who were treated with HSCT (cc, 0,905). Similar correlation was found when the number of experts was compared with those treated with HSCT. However, the number of patients treated with subcutaneous Ig (SCIG) only slightly correlated with the number of experts (cc, 0,489) and no correlation was found between the number of centers and patients on SCIG (cc, 0,174).

**Conclusions:**

1) this is the first study describing major diagnostic and treatment parameters of IEI care in countries of the JP; 2) the data suggest that the JP had tremendous impact on the development of IEI care in ECE; 3) our data help to define major future targets of JP activity in various countries; 4) we suggest that the number of IEI centers and IEI experts closely correlate to the most important treatment parameters; 5) we propose that specialist education among medical professionals plays pivotal role in increasing levels of diagnostics and adequate care of this vulnerable and still highly neglected patient population; 6) this study also provides the basis for further analysis of more specific aspects of IEI care including genetic diagnostics, disease specific prevalence, newborn screening and professional collaboration in JP countries.

## Introduction

Despite tremendous progress in the field over the past decades, primary immunodeficiency disorders (PIDs) also referred to as inborn errors of immunity (IEI) still represent a neglected area of medicine (1–3). Basic research into the field is concentrated in a small number of centers in the USA, Western Europe, Japan, China and Australia. Incidence and prevalence data are either not available or vary remarkably in different countries (4–6; this study). Legal restrictions and diagnostic difficulties in many countries make patient registries incomplete and unreliable (4). Immunology education at both graduate and postgraduate levels is focused mostly on autoimmunity and allergic diseases neglecting IEIs. Experts with the allergy-immunology license exam may have limited knowledge on IEIs especially their mechanistic and genetic dimensions.

More than 450 IEIs have been described but many of them only in a few patients and families and data were obtained mostly from mice studies (7–11). Limitations to publish rare IEI cases in medical journals hamper the accumulation of substantial amount of clinical data. Primarily IEI-focused medical journal, like the J Clin Immunol has only recently been established (12). Most hematology journals are reluctant to take IEI cases even with hematological phenotype of the patient (personal communication). IEI continues to receive negligible attention by governmental agencies and well-functioning, advanced IEI centers may suffer from brutal attack from unprofessional institutional leaders (JP Book 2015, pp. 56-57; www.thejpnetwork.com).

Despite the above listed difficulties and drawbacks, the field has been growing and progressing largely due to the development of molecular immunology and genetics (13–15). Since the characterization of the first IEI in the early fifties a large number of patients with new disease entities were described on immunological bases. Since the eighties, IEIs started to become widely known as a genetically defined group of diseases caused by single gene mutations (13). Very often, however, high tech genetic studies result in the discovery of new diseases only in one or a few individuals or families, and diagnosis of new cases are limited by the lack of available genetic assays (2, 16). The gap developing between advanced molecular genetic knowledge in leading centers and the limited accessibility of IEI diagnosis and treatment has to be closed despite the above listed obstacles. Novel educational and collaboration programs have been created to spread knowledge and to start IEI-focused medical care. One of these programs which can be considered as a prototype is the J Project (JP) started in 7 Eastern and Central European (ECE) countries in 2004 and covering now a large area of 32 countries in Eastern and Central Europe (ECE), Asia and Africa (16–23). In this paper we report major diagnostic and treatment parameters of IEI care that had been achieved by the end of 2021 in countries involved in the JP.

## Results and discussion

### JP meetings

**Figure 1:**
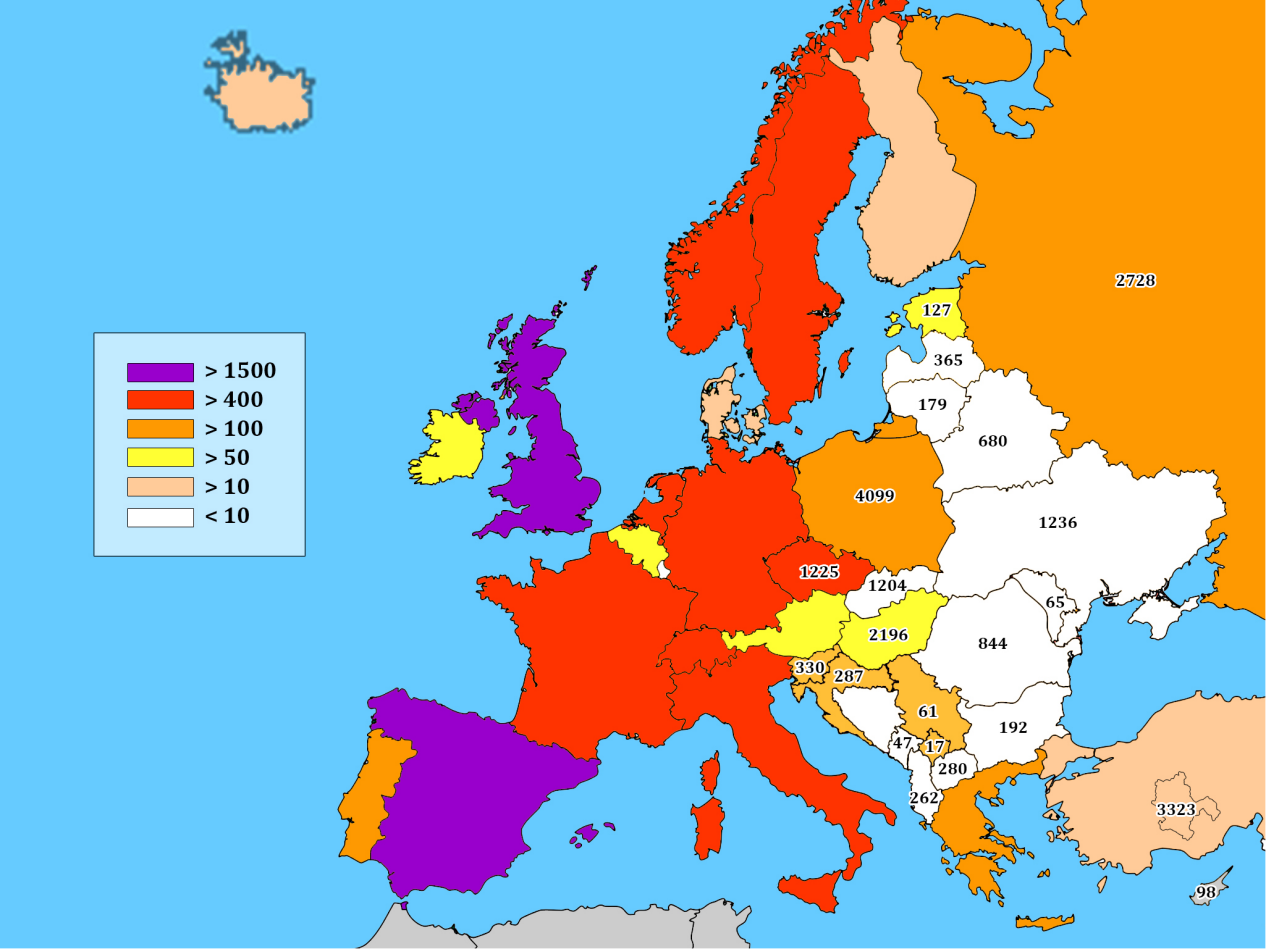
Number of patients with inborn errors of immunity reported from Eastern and Central Europe (ECE) to the ESID Registry in 2000 was less than 10 (see color coding and the scale on the left). Number of patients reported to this J Project survey at the end of 2021 is shown by numbers in ECE countries. For more details see **Table 1**.

**Figure 2:**
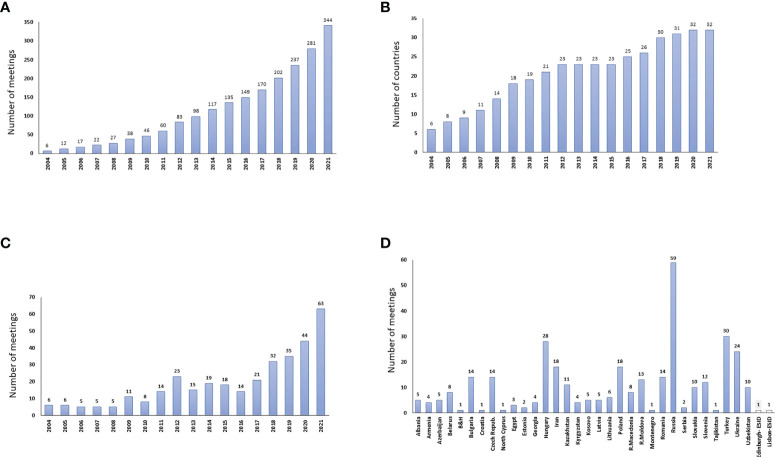
**(A)** shows the cumulative number of J Project (JP) meetings organized in Eastern and Central Europe (ECE), Asia and part of Africa. The average number of meetings per country was 10.75 in 2021. **(B)** shows the number of countries participating in the JP between 2004 and 2021 reaching 32 by 2020. **(C)** shows the number of JP meetings organized yearly over 18 years. A remarkable increase of meeting number occurred over the past 4 years which was not directly related to the number of participating countries. This is indicated by the difference between 2020 and 2021 when the JP country number was equal. **(D)** shows the cumulative number of JP meetings in participating countries. The largest number of meetings in Russia is in concert with the population estimated 138,000,000 in this country. On the other hand, the number of meetings in Hungary (estimated population, 9,600,000) was about half of that in Russia. Further, the same number of meetings were organized in Bulgaria and Czech Republic than in Romania with a population of 20,000,000 and Republic of Moldova (2,600,000) or Slovenia (2,200,000) suggesting that the activity and interest maybe more important than the size and population of the country. Unfortunately, there are countries with permanently low number of meetings including Croatia, Bosnia & Herzegovina, Estonia, Serbia) despite longer-term membership in the JP network (see also **Supplementary Table 1**).

At the turn of the millennium many countries in ECE reported less than 10 patients to the European Society for Immunodeficiency Registry (ESID-R) (24; **Figure 1**). Thus, taken 10 countries (with the exception of Bosnia & Herzegovina, Montenegro and Kosovo), the cumulative number of patients increased from max 100 to 5307 by the end of 2021 (**Figure 1**). A conceptual IEI-focused professional meeting series was started in 2004 and reached measurable results even in countries with low socioeconomic conditions including the Rep. of Moldova, Rep. of North Macedonia, Albania, and Kosovo (11, 25, 26). Over the past 18 years, 344 IEI-focused conferences were organized (**Supplementary Table 1** and **Figure 2A**). These events have resulted in a remarkable progress in diagnosis and treatment of patients with IEI. More and more countries, first in Central Europe, next in Eastern Europe, and in 2009, Iran, Turkey and Egypt joined the JP and we have now 32 so-called JP countries (**Figure 2B**). The number of JP meetings increased from 6 per year in 2004 and 2005 to 44 and 63 per year in 2020 and 2021, respectively (**Figure 2C** and **Supplementary Table 1**). The cumulative number of meetings varied from 1 to 59 in various countries reflecting, at least in part, the population of the respective countries (population and meeting number, correlation coefficient, cc, 0,72) (**Figure 2D**).

### JP congresses

The vast majority of JP meetings were regional or national events organized by local opinion leaders. Three JP Congresses were also organized, traditionally in Antalya, Turkey by I Reisli, leader of the J Daughter Anatolia Project, in 2014, 2016, and 2019. In 2014, not only the first JP Congress but the 10^th^ anniversary of the establishment of the JP and the 100^th^ JP events that had been organized were celebrated. These JP Congresses with participation of the most prominent IEI researchers from all over the world were devoted to discuss newly published or unpublished novel primary immunodeficiencies in order to stimulate further clinical research in JP countries and promote collaboration. Importantly, these congresses provided excellent occasions to present novel clinical observations of colleagues from JP countries. In 2016 and 2019 we issued two declarations for patients with primary immunodeficiencies which we referred to as “Konya Declarations” (27).

### The JP Steering Committee

To coordinate the activity of the JP, the JP Steering Committee (SC) was established in 2010; following this year SC meetings were regularly organized mostly in Budapest, Hungary, or at the time of ESID congresses (Edinburgh and Lisbon in 2017 and 2018, respectively) (**Supplementary Figure 1**). These SC meetings outlined previous achievements and future programs of the JP including joint clinical research which were published in reasonable international journals (28–33). An SC meeting to remember was the one in March 2019. By this time the JP was about to consider to become a new IEI society. This meeting was attended by leaders of the ESID (I Meyts, F Candotti, A Cant) and at the end of the long discussions of pros and contras we decided to go on further as a network and collaborate rather than compete with ESID.

### Opening the scope of education and research: The COVID pandemic

The JP has been continually changing ever since its creation in 2004, recognizing the increasing need for physician education and clinical research collaboration initially in Central Europe, then in Eastern Europe, and subsequently elsewhere, most recently in Central Asia and Far-East Russia. This evolution based on the recognition of these needs has resulted in very sensitive changes and rearrangements of the JP program throughout Eurasia, as reflected in the annual editions of JP booklets and our regularly updated website (www.thejpnetwork.com). The prime focus of the collaboration has shifted from clinical education to genetics teaching.

Due to the dynamic progress and popularity of the JP, the area for which we hold responsibility had extended to the Pacific by 2000, and is bordered now by two oceans and eight seas (**Supplementary Figure 2**). This huge geographic dimension was never anticipated in our initial plans, when we established the Project in the Carpathian region of Europe, with no intentions to expand it elsewhere. In the JP book for 2019, we expressed our enthusiasm concerning the success with which knowledge of next-generation sequencing technologies had been disseminated, making it possible to define the genetic basis of more PIDs. However, in 2020 events took an unexpected turn, forcing the world to face new challenges. The very existence of humanity had been threatened from a viral disease caused by SARS-Cov-2. Most research laboratories, centers and institutions have changed direction and focused their research on studies of the mechanisms, prevention and treatment of COVID-19, the most severe form of coronavirus disease. The JP has joined forces with one of the most progressive PID research laboratories, led by J-L Casanova at the Rockefeller University and in Paris, and we have agreed to extend participation in the COVID-19 research of this laboratory to the whole area of the JP (34). The JP has been taking part in this research by establishing participating centers all over Eurasia and increasing awareness of unusual COVID-19 cases at JP meetings.

Organization of the JP was strongly supported from the beginning by the ESID and the Jeffrey Modell Foundation (JMF) as well as by grants from a few pharmaceutical companies (18). Based on SC decision, the JP meeting organization has been coordinated by the Foundation for Children with Immunodeficiencies since 2014.

### Patients diagnosed with IEI in JP countries

**Figure 3:**
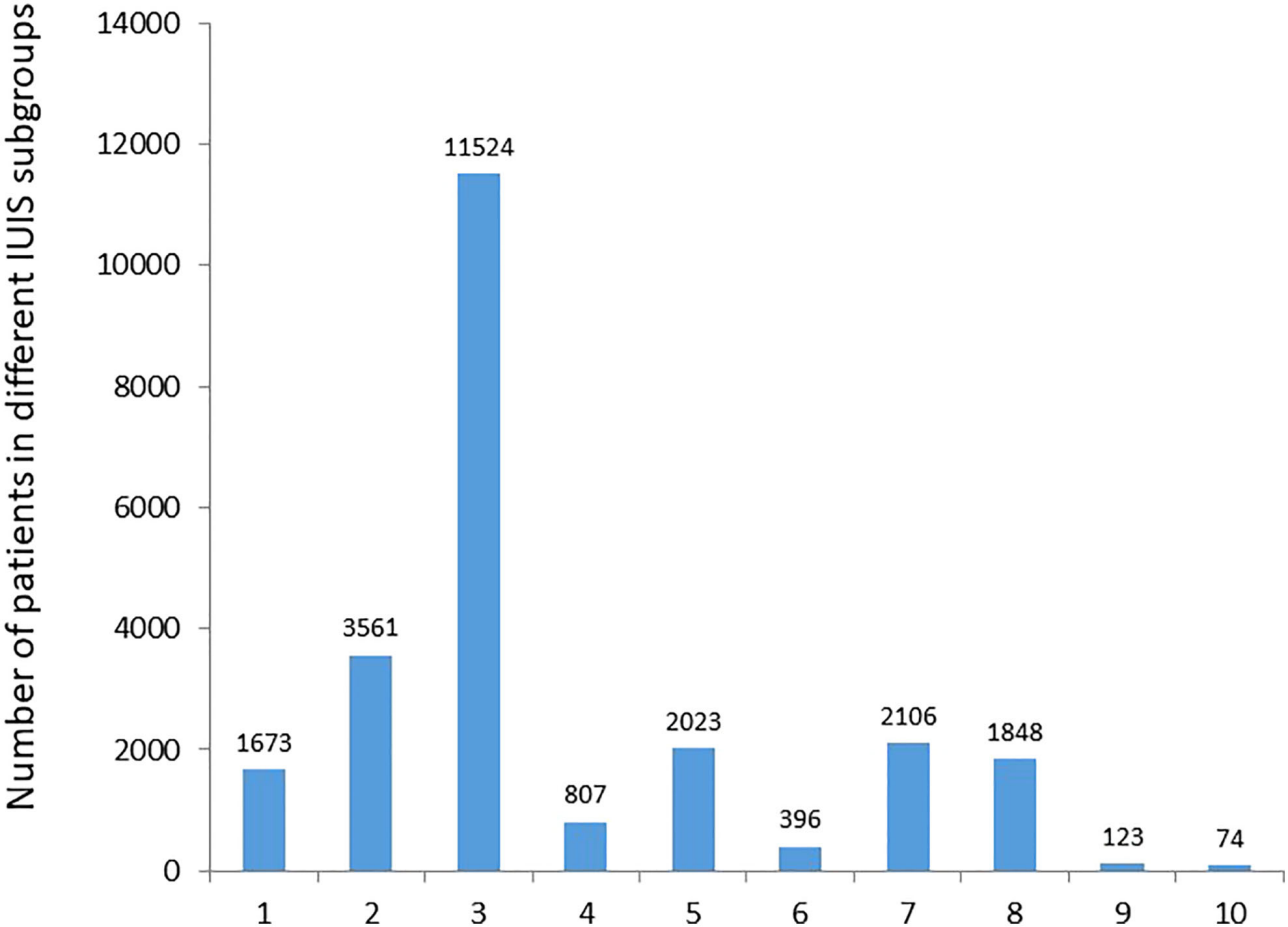
The total number of patients reported from 30 countries to various IUIS subgroups. The most common subgroups were predominantly antibody deficiency (11510) and combined immunodeficiencies with associated or syndromic feature (3557). The number of patients with periodic fever syndrome does not include patients reported from Armenia (3151 pts) to avoid unproportional presentation (see **Table 1**). The number of patients with no definitive IEI diagnosis was 734 representing 2,95% of the total number of 24,862 patients.

After 18 years of JP educational activity and published research we decided to put together basic parameters of patient care. To this end, questionnaires about diagnosis and treatment of patients were sent out to center leaders who were requested to fill them out and return with comments. This parameters survey was intended to be a kind of snapshot on what we had achieved and where we should be going to. Thus, specific questions about age, gender, disease duration and severity of illness and similar details were not included. We believe that JP centers should use primarily the ESID-R for entering patients data in more detail. Altogether, 24,879 patients with various IEIs were reported (**Table 1**, **Figure 3** and **Supplementary Figure 3**). Classification was made according to the IUIS committee of IEI: 1) Immunodeficiencies affecting cellular and humoral immunity; 2) Combined immunodeficiencies with associated or syndromic features; 3) Predominantly antibody deficiency; 4) Diseases of immune dysregulation; 5) Congenital defects of phagocyte number or function; 6) Defects in intrinsic and innate immunity; 7) Auto-inflammatory disorders; 8) Complement deficiencies; 9) Bone marrow failure; 10) Unclassified inborn error of immunity or IEI phenocopies. Most patients had predominantly antibody deficiency (11,524; 46,32%) followed by patients with combined immunodeficiencies with associated or syndromic feature (3,561; 14,31%). The percentages of patients with bone marrow failure and phenocopies of IEI were less than 1% each, respectively. These data were reported from 28 JP countries and two centers representing an estimated population of 506 567 565 (**Table 1****).** Patients from Bosnia & Herzegovina and Tajikistan were not reported; the population of these two countries is estimated to be 12,42 million. The two countries that participated in the survey by reporting patients from JP centers were Turkey (Konya Center) and Egypt (Cairo University Center). Based on individual data, the prevalence of IEI in JP countries varied between 0.07 in Uzbekistan and 41.54 in Konya Center, Turkey, with an average prevalence of 4.9 (**Table 1**). The number of centers per country varied from 1 to 107 and correlated well with the population of the country (cc, 0,90) (**Table 2**).

**Table 1:** Reported patients with inborn errors of immunity from J project countries/centers.

Country	Inborn errors of immunity according to the IUIS classification^*^											All	Estimated population	Patients /10^5^
	1	2	3	4	5	6	7	8	9	10	UD			
1. Albania	6	17	73	5	134^§^	4	12	2	5	1	3	262	2 829 741	9.26
2. Armenia	0	0	4	0	4	0	(3151)	6	0	0	0	14	2 963 000	0.47
3. Azerbaijan	13	36	45	22	6	0	0	0	0	0	13	135	10 157 000	1.33
4. Belarus	47	173	198	51	34	3	4	85	24	3	58	680	9 600 000	7.08
5. Bosnia&H	No patients were reported because of government regulation													
6. Bulgaria	10	33	86	4	10	0	21	11	0	17	0	192	6 916 548	2.76
7. Croatia	18	70	115	6	25	10	1	28	4	1	9	287	3 888 529	7.38
8. Czech Rep	22	253	657	17	30	3	22	213	0	8	0	1225	10 700 000	11.45
9. N Cyprus	0	0	96	0	0	0	0	0	0	0	2	98	475 442	20.61
10. Egypt^@,a^	294	72	88	157	195	81	487	11	20	0	64	1469	18 000 000	8.16
11.Estonia	2	7	96	0	6	3	0	7	0	0	6	127	1 328 439	9.56
12. Georgia	1	0	8	0	1	0	92	0	0	0	20	122	3 979 765	3.07
13. Hungary	82	77	1304	69	59	26	49	360	13	4	153	2196	9 689 000	22.66
14. Iran	368	521	903	63	524	125	490	62	0	0	0	3056	84 000 000	3.64
15. Kazakhstan	9	7	142	5	8	0	3	24	0	6	0	204	19 135 477	1.07
16. Kyrgyzstan	0	0	0	0	0	0	0	0	0	0	9	9	6 592 000	0.14
17. Kosovo	1	4	8	0	4	0	0	0	0	0	0	17	1 935 000	0.88
18. Latvia	2	57	253	4	18	2	12	11	5	0	1	365	1 890 000	19.31
19. Lithuania	3	8	104	1	3	2	2	29	2	6	19	179	2 795 000	6.40
20. Macedonia	10	41	162	2	18	2	27	7	0	0	11	280	2 083 254	13.44
21. Moldova	3	11	50	1	0	0	0	0	0	0	0	65	2 597 000	2.50
22. Montenegro	1	6	4	1	28	0	1	4	0	2	0	47	621 718	7.56
23. Poland	169	879	2156	59	283	42	182	132	8	12	177	4099^#^	38 091 094	10.76
24. Romania	18	44	408	21	150	7	46	112	17	0	21	844	19 030 136	4.43
25. Russia	368	591	699	196	262	43	221	342	0	6	0	2728	145 478 097	1.87
26. Serbia	2	9	32	2	5	0	0	9	2	0	0	61	8 683 801	0.70
27. Slovakia	22	116	416	21	20	11	285	257	4	1	51	1204	5 449 270	22.09
28. Slovenia	17	82	54	37	39	6	18	74	3	0	0	330	2 080 000	15.87
29. Tajikistan	No patients were reported because of developmental issues													
30. Turkey^@,b^	133	166	2724	16	66	16	99	16	10	7	70	3323	8 000 000	41.54
31. Ukraine	46	276	631	47	91	10	26	46	6	0	57	1236	43 342 300	2.85
32. Uzbekistan	6	5	8	0	0	0	6	0	0	0	0	25	34 235 954	0.07
Summary	1673	3561	11524	807	2023	396	2106	1848	123	74	744	24 879	506 567 565	Average: 4.9
Percentage	6.72	14.31	46.32	3.24	8.13	1.59	8.46	7.43	0.49	0.30	2.99	100	–	–

*Tangye et al, JoCI, 2020; ^§^These data include patients with hypo-IgA in the paripheral blood; ^@^Data were reported from ^a^Cairo and ^b^Konya centers; ^#^Malgorzata P, Bernatowska E. Eur J Pediatr 2016; 175(8):1099. UD, Unclassified disease; H, Herzegovina; N, North; IUIS, International Union of Immunological Societies.

**Table 2:** Diagnostic and treatment options in JP countries/centers.

Country	Available diagnostic parameters						Treatment options			
	Ct^1^	Exp^2^	ImmChem^3^	FlowCyt^4^	TGS^5^	NGS^6^	IVIG^7^	SCIG^8^	HSCT^9^	Other treatments
Albania	1	5	Y	Y	N	N	10	0	3	IS
Armenia	1	2	Y	Y	Y	Y	8	0	3	G-CSF
Azerbaijan	1	5	Y	Y	N	N	28	0	3	Thymus hormone
Belarus	2	8	Y	Y	Y	Y	37	29	33	G-CSF, IFN-γ, IS, biological therapy
Bosnia & H	No data were reported									
Bulgaria	1	15	Y	Y	Y	Y	5	49	4	Biological therapy
Croatia	2	6	Y	Y	Y	N	53	62	21	G-CSF, biological therapy
Czech Rep	17	42	Y	Y	Y	Y	168	301	86	Thymus transplantation, biological therapy
N Cyprus	2	2	Y	Y	N	N	12	0	0	IS, biological therapy
Cairo Center	1	10	Y	Y	Y	Y	62	0	25	G-CSF, IS, biological therapy
Estonia	2	6	Y	Y	Y	Y	49	9	5	IS, biological therapy
Georgia	1	1	Y	Y	N	N	0	0	0	IS, biological therapy
Hungary	7	17	Y	Y	Y	Y	430	160	70	G-CSF, IFN-γ, IS, virus specific T cell therapy
Iran	30	100	Y	Y	Y	Y	563	0	175	G-CSF, IFN-γ
Kazakhstan	2	12	Y	Y	Y	N	9	92	3	IFN-γ, G-CSF, anti-TNF-α
Kyrgyzstan	1	1	N	N	N	N	0	0	0	IS
Kosovo	1	2	N	N	N	N	2	1	1	IS
Latvia	2	7	Y	Y	Y	Y	14	20	15	IL-1RA, anti-TNF-α
Lithuania	2	11	Y	Y	Y	Y	4	50	2	anti-TNF-α, IFN-γ, G-CSF
N Macedonia	1	3	Y	Y	Y	Y	30	2	2	G-CSF, IS, biological therapy
R Moldova	1	5	Y	Y	Y	N	5	0	1	G-CSF
Montenegro	1	2	Y	Y	N	N	2	0	5	IS
Poland	19	160	Y	Y	Y	Y	331	510	320	IS
Romania	8	13	Y	Y	Y	Y	42	7	8	G-CSF
Russia	107	168	Y	Y	Y	Y	2101	11	492	IS, CSFs, ADA, biological therapy
Serbia	4	6	Y	Y	Y	Y	56	5	15	G-CSF, IS
Slovakia	3	12	Y	Y	Y	Y	60	200	39	IFN-γ, biological therapy, thymus transplantation
Slovenia	1	8	Y	Y	Y	Y	30	16	32	Thymus transplantation, virus specific T cell therapy
Tajikistan	No data were reported									
Turkey	28	44	Y	Y	Y	Y	1500	400	250	ADA, IFN-γ, gene therapy
Ukraine	7	16	Y	Y	N	N	160	84	47	G-CSF
Uzbekistan	2	4	N	Y	N	N	12	–	–	IS
Summary (Y)	260	690	(27)	(28)	(21)	(18)	5693	1879	1480	–
Mean + SEM	9+3.8	23.9+8	–	–	–	–				–

^1^N^o^ of centers; ^2^N^o^ of experts; ^3^Immunochemistry (yes or no); ^4^flow cytometry (yes or no); ^5^targeted gene sequencing (yes or no); ^6^new generation sequencing (yes or no); ^7^N^o^ of patients receiving intravenous immunoglobulin; ^8^N^o^ of patients receiving subcutaneous immunoglobulin; ^9^N^o^ patients who received hematopoietic stem cell therapy; IS, immunosuppression; G-CSF, granulocyte colony stimulation factor; IFN- γ, interferon-gamma; TNF-α, tumor necrosis factor alpha; IL1-RA, interleukin-1 receptor antagonist; ADA, adenosine deaminase.

**Figure 4:**
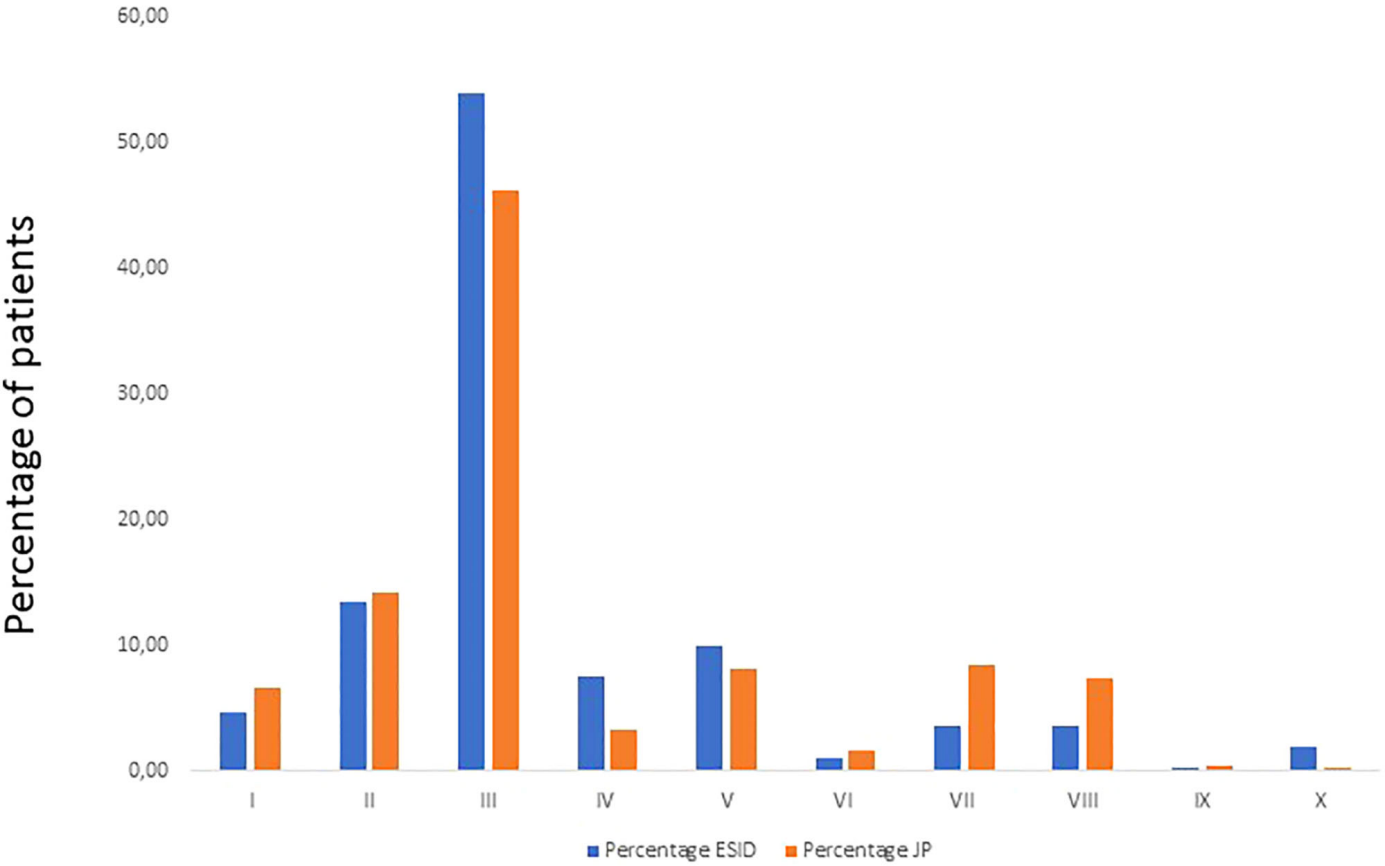
Comparable percentages of patients in different subgroups reported to the ESID-R (left columns) and the J Project (right columns). Despite slight differences in a few subgroups (subgroups 3, 4, 7 and 8), these data indicate similarly wide range of diagnosis of patients with different disease groups.

**Figure 5:**
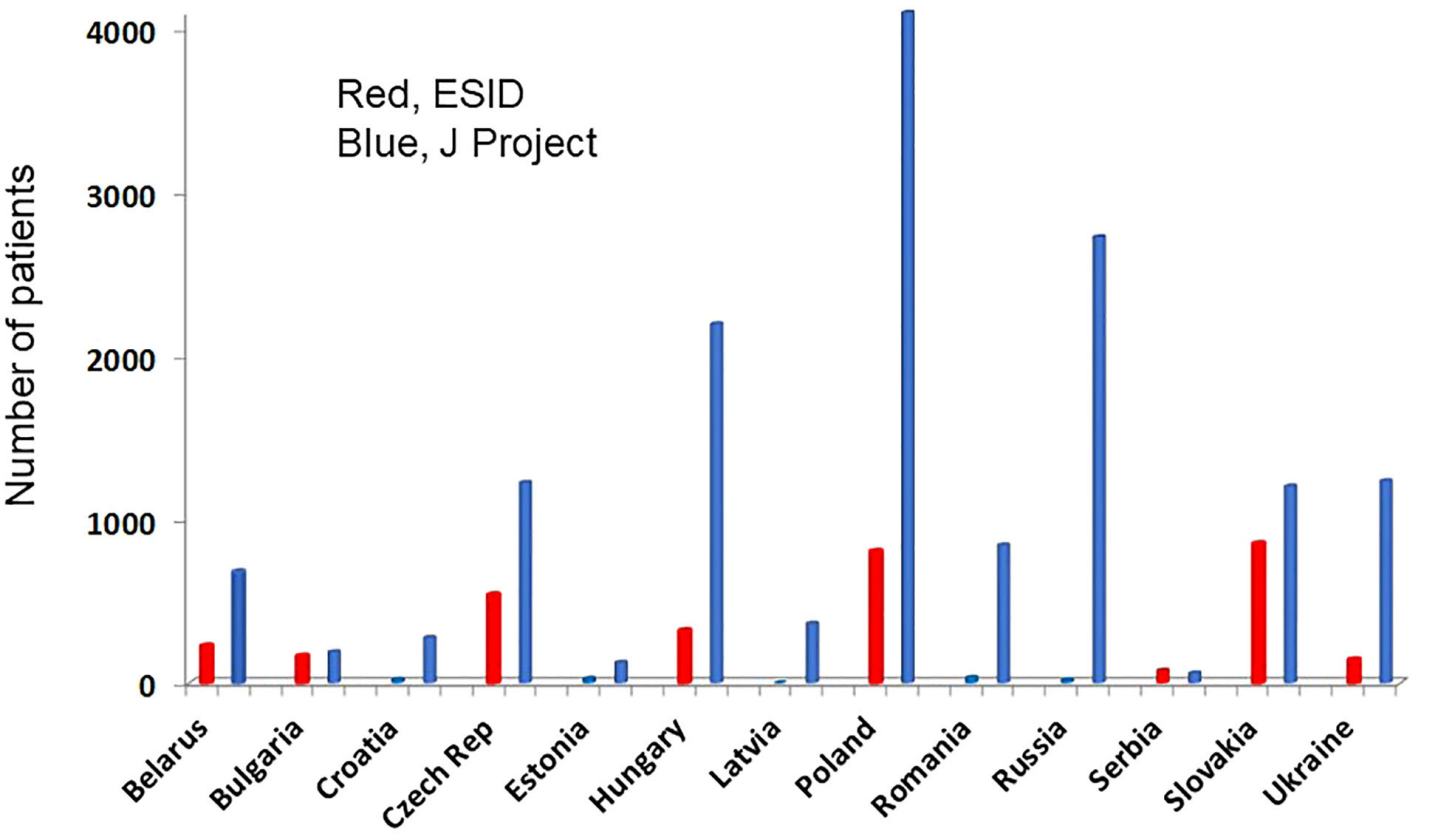
Total number of patients reported to the ESID-R (left columns, red) and the J Project (JP; right columns, blue) are shown. Such data were available only from 13 of the 32 JP countries indicating the lack of appropriate reporting activity. In addition, the total number of patients reported to the ESID-R was 3226 in contrast to the 15,234 patients reported in this survey.

We compared the percentage of patients in different IUIS subgroups reported to the ESID-R and the J Project. The data showed that the percentage of reported patients was comparable with only mild differences in subgroups III and IV, which were slightly higher in ESID-R, and in subgroups VII and VIII, which were somewhat higher among patients reported to the JP (**Figure 4**). These data are promising and suggest comparable attention to the wide range of IEI in the Western and Eastern parts of Europe. However, diagnosis of specific diseases, especially recently described IEIs may be completely different and should be analyzed in future. Importantly, the total number of patients reported to this survey was remarkably higher than those reported to the ESID-R in 13 countries (**Figure 5**). These data suggest that reporting activity to the ESID-R should be increased in JP centers to make the ESID-R a reliable database and a solid source of information about the widest range of IEI in both Western and Eastern Europe. Nineteen JP countries did not report at all to the ESID-R

In the beginning of JP activity, patients in centers were diagnosed primarily with antibody deficiencies. This is well exemplified by North Cyprus that joint the JP in 2019 and reported only patients with antibody deficiencies even in 2021 (**Table 1**). Also, Uzbekistan joining the JP in 2018 reported patients that fall only in 4 diseases’ groups of IEI (**Table 1**). Countries with the highest prevalence value (Turkey, Hungary and Slovakia), however, reported a full spectrum of IEI patients suggesting a wide range of diagnosis. Similar data were observed in countries like Albania, Belarus, Lithuania and Poland with prevalence between 6.4 and 10,76 (**Table 1**).

**Figure 6:**
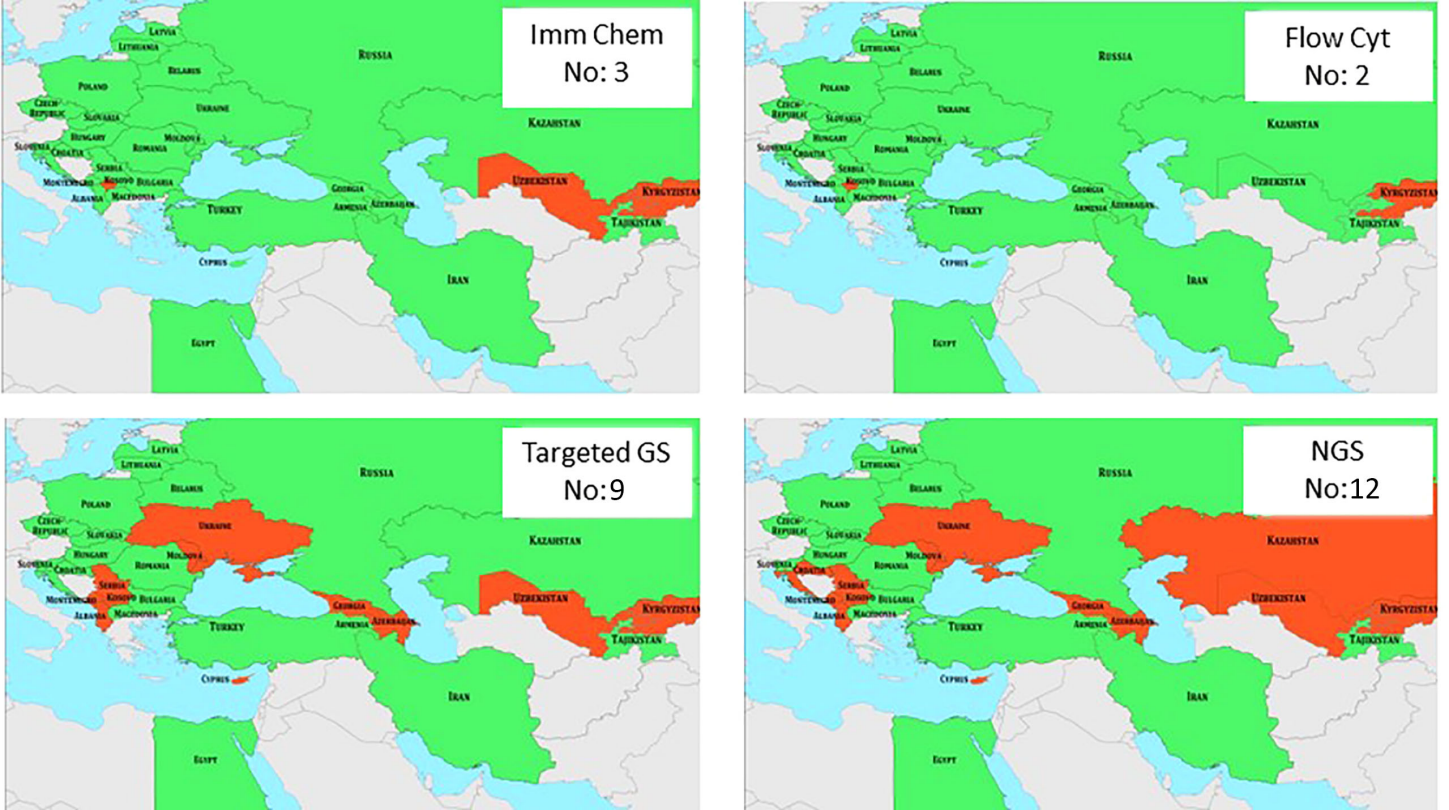
Availability of basic diagnostic parameters in J Project countries is shown in green. Red indicates the lack of various parameters. Numbers in insets indicate the number of countries that are missing various diagnostic measures. Imm Chem, immunochemistry; Flow Cyt, flow cytometry; GS, gene sequencing; NGS, new generation sequencing.

Searching for basic diagnostic parameters revealed the availability of both immunochemistry and flow cytometry in 27 and 28 countries, respectively, but targeted gene sequencing and next generation sequencing was available only in 21 and 18 countries (**Table 2** and **Figure 6**). These parameters are generally available in Central Europe but immunochemistry is still missing in 3 countries (Kosovo, Uzbekistan, Tajikistan) and flow cytometry in two countries (Kosovo and Kyrgyzstan). Genetic analysis is unavailable in Ukraine, a few South European countries, two Caucasian countries, and most Central Asian countries (**Table 2** and **Figure 6**). No data are available from Bosnia & Herzegovina and Tajikistan. Together, these data suggest that further development of diagnostics is needed in Central Asia through more focused educational activity about the relevant genetic diagnosis in patients and families.

### Treatment parameters

The number of experts ranged between 1 and 168 and there was also a strong correlation with the country populations (cc, 0,81; **Table 2**). Most patients diagnosed first suffered from antibody deficiency and were treated with intravenous immunoglobulins (IVIG) and later with subcutaneous immunoglobulin (SCIG) preparations. These treatment schedules were completed with hematopoietic stem cell transplantation (HSCT) in patients having both T cell and B cell immunodeficiencies even without precise genetic analysis. A wide range of other treatment options in IEI patients were also reported including a variety of medicines under the heading of biological therapy (**Table 2**).

**Figure 7:**
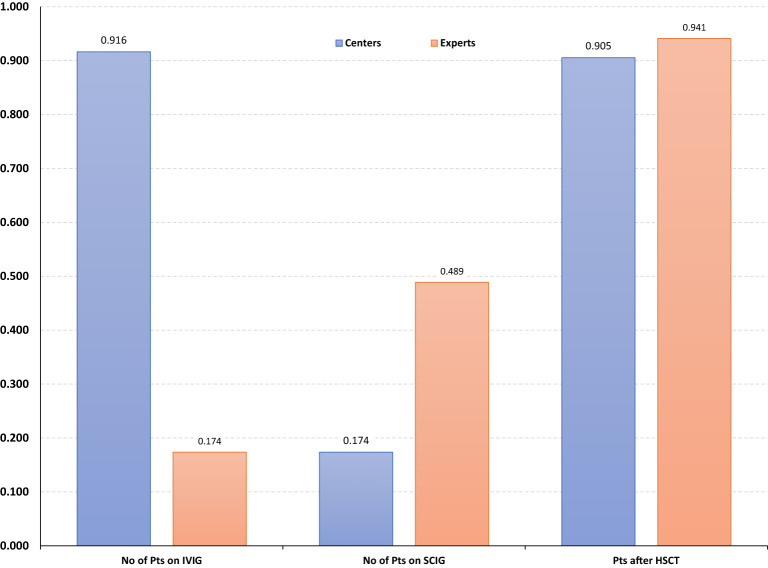
Correlations between the number of primary immunodeficiency (PID) centers and PID experts with the replacement of intravenous immunoglobulin (IVIG) or subcutaneous Ig (SCIG) or the number of hematopoietic stem cell transplantation (HSCT) performed in various countries. Data show that higher number of centers and experts favored the treatment with IVIG and HSCT but not with SCIG.

By the end of 2021, immunoglobulin substitution had been provided to 7,572 patients (5693 intravenously) and 1480 patients had received HSCT (**Table 2**). The number of IEI centers and experts were 260 and 690, respectively. We found high correlation between the number of PID centers and patients treated with IVIG (cc: 0,916) and with those who were treated with HSCT (cc: 0,905) (**Figure 7**). Similar correlation was found when the number of experts was compared with the number of patients treated with HSCT. However, the number of patients treated with SCIG only slightly correlated with the number of experts (cc: 0,489) and no correlation was found between the number of centers and patients on SCIG (cc: 0,174) (**Figure 7**). Although the total number of patients receiving IVIG was about three times higher, in eight countries more patients received SCIG (**Table 2**). In three countries (Lithuania, Bulgaria, and Kazakhstan) ten times more patients received SCIG compared to that of IVIG. In addition to several advantages of SCIG over IVIG, this could be due to the SARS-CoV-2 pandemic (35).

## Conclusion

Several papers have been published before about the educational activity of the JP and the spread of the program in Eurasia (18, 30). In this study we first describe major diagnostic and treatment parameters of IEI care in countries of the JP after it had “grown up” and reached its 18 years in 2021 and outlined the progress we have made in a very important field of molecular and clinical medicine. We propose here that the JP has had remarkable impact on the development of IEI care and research in ECE and part of Asia. This ambitious project with the leadership of the JMF center established in 2004 at the Department of Infectiology and Pediatric Immunology in Debrecen, Hungary, was originally focused on a small area of Central Europe referred to as the Carpathian Euro region (**Supplementary Figure 4**). Due to outstanding ambition and support from international organizations and foundations, it has been permanently spreading across political, cultural and religious borders of Eurasia (24). We have come so far from our original plan and the progress we have made achieved the attention of the professional community worldwide. Similar successes were achieved and must be mentioned here in various parts of Asia, Africa, and South America (36, 37). Our data not only give hope to patients with PID but help to define major future targets of IEI awareness campaign and research in different continents. Importantly, the data presented here suggest that the number of IEI centers and IEI experts closely correlate to the most important treatment parameters, i.e. IVIG substitution and HSCT. We provide evidence that specialist education among medical professionals plays pivotal role in assuring diagnosis and adequate care in this vulnerable and still highly neglected patient population. This study also provides the basis for further analysis of more specific aspects of IEI care including genetic diagnostics, disease-specific prevalence and newborn screening as well as clinical research collaboration in J Project countries. Genetic testing of patients included in this study had not been requested because of the heterogenicity of diagnostic facilities in JP countries over the 18 years. This may represent a limitation of this study, but more importantly, it points to one of the focus of further research in the J Project.

The JP is an inspiring story for future immunologists worldwide and for the next generation of the Project in Eurasia. The secret sets of the success we described here is double: No 1: professional devotion, love of patients and research, and collaboration in any possible way we can; No 2: whatever we have achieved, there is so much more to do: more active participation at the advanced IEI communities like ESID, Latin American Society for Immunodeficiencies (LASID), African Society for Immunodeficiencies (ASID), and Clinical Immunology Society, improved quality of research publications, more organized national IEI patient care and data reporting to international databasis.

## Data availability statement

The original contributions presented in the study are included in the article/**Supplementary Material**. Further inquiries can be directed to the corresponding author.

## Ethics statement

This study was approved by the Ethical Committee of the University School of Medicine, Debrecen, Hungary (ETT HRB 5975/2014/EHR and DE OEC RKEB/IKEB 3851-2013). Written informed consent to participate in this study was provided by the participants’ legal guardian/next of kin.

## Author contributions

IL performed all statistical analysis and holds the first authorship. GKi provided ESID Registry data. LM formulated the research goals and wrote the final draft. All other authors conducted clinical research and patient care and approved the submitted version. ASe, NG, NR, EB, MPa, ASh, PC, MJ, IR, ABo, TA, and LM share last authorship for their unique contribution to this work. All authors contributed to the article and approved the submitted version.

## Acknowledgments

The authors thank all professionals not listed among authors who took part in the complex care of patients described in this study. We thank I Meyts, F Candotti, J Litzman, L Malinauskiene, E Gasiuniene and V Mulaosmanovic for helpful discussion. We are grateful to László Maródi, Jr, for composing the J Project Song (https://www.youtube.com/watch?v=5z_z1sHmRnY).

## Conflict of interest

The authors declare that the research was conducted in the absence of any commercial or financial relationships that could be construed as a potential conflict of interest.

## Publisher’s note

All claims expressed in this article are solely those of the authors and do not necessarily represent those of their affiliated organizations, or those of the publisher, the editors and the reviewers. Any product that may be evaluated in this article, or claim that may be made by its manufacturer, is not guaranteed or endorsed by the publisher.

## Supplementary material

Supplementary Figure 1 The first J Project (JP) Steering Committee meeting in 2010, chaired by (from L to R) I Reisli, M Serban, L Maródi (center), I Tuzankina, M Pac, and A Bondarenko. Delegates from all JP member countries, 19 at that time, attended the meeting in order to discuss previous achievements and future challenges of the JP.

Supplementary Figure 2 Geographic extension of the J Project by 2020 which included 32 countries and 6 J Daughter (JD) Project regions chaired by N Rezaei (JD Persia, 2009), I Reisli (JD Anatolia, 2009), A Elmarshafi and N Galal (JD Egypt, 2009), I Tuzankina (JD Siberia, 2010), E Kovzel (JD Central Asia, 2012), and E Tcyvkina (JD Far East Russia, 2019).

Supplementary Figure 3 Pie diagram showing the percentages of various inborn errors of immunity subgroups of patients reported to the J Project.

Supplementary Figure 4 The first 8 J Project meetings organized in Central Europe.
