# Supplementary figures and images for: Care of patients with inborn errors of immunity in thirty J Project countries between 2004 and 2021
Source: Front Immunol. 2022 Dec 16;13:1032358. doi: 10.3389/fimmu.2022.1032358 (PMC9809467; doi:10.3389/fimmu.2022.1032358)

Suppl. Fig. 1


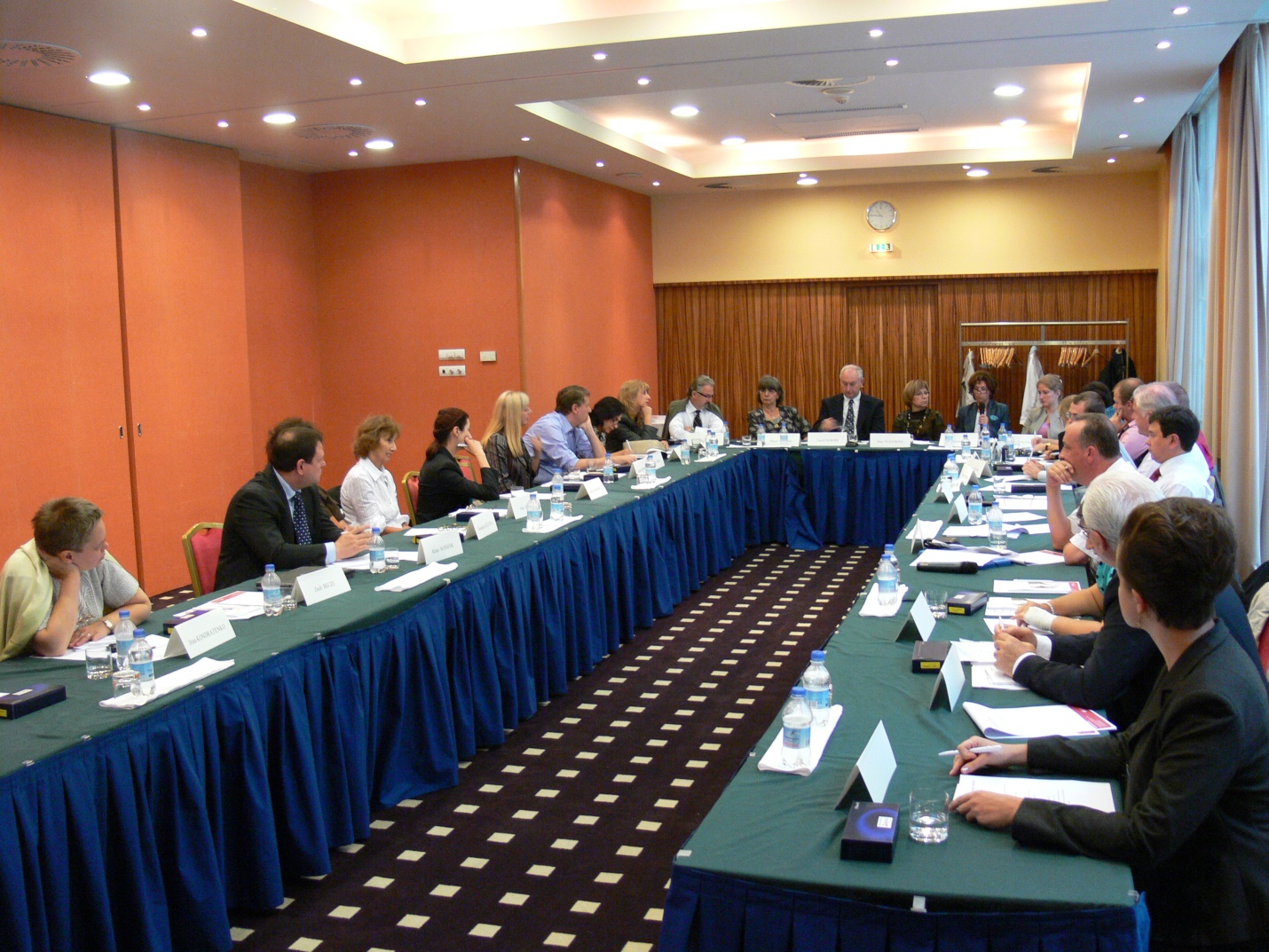

Supplement: Supplementary Figure 1 — The first J Project (JP) Steering Committee meeting in 2010, chaired by (from L to R) I Reisli, M Serban, L Maródi (center), I Tuzankina, M Pac, and A Bondarenko. Delegates from all JP member countries, 19 at that time, attended the meeting in order to discuss previous achievements and future challenges of the JP. [file DataSheet_1.docx]

Suppl. Fig. 2


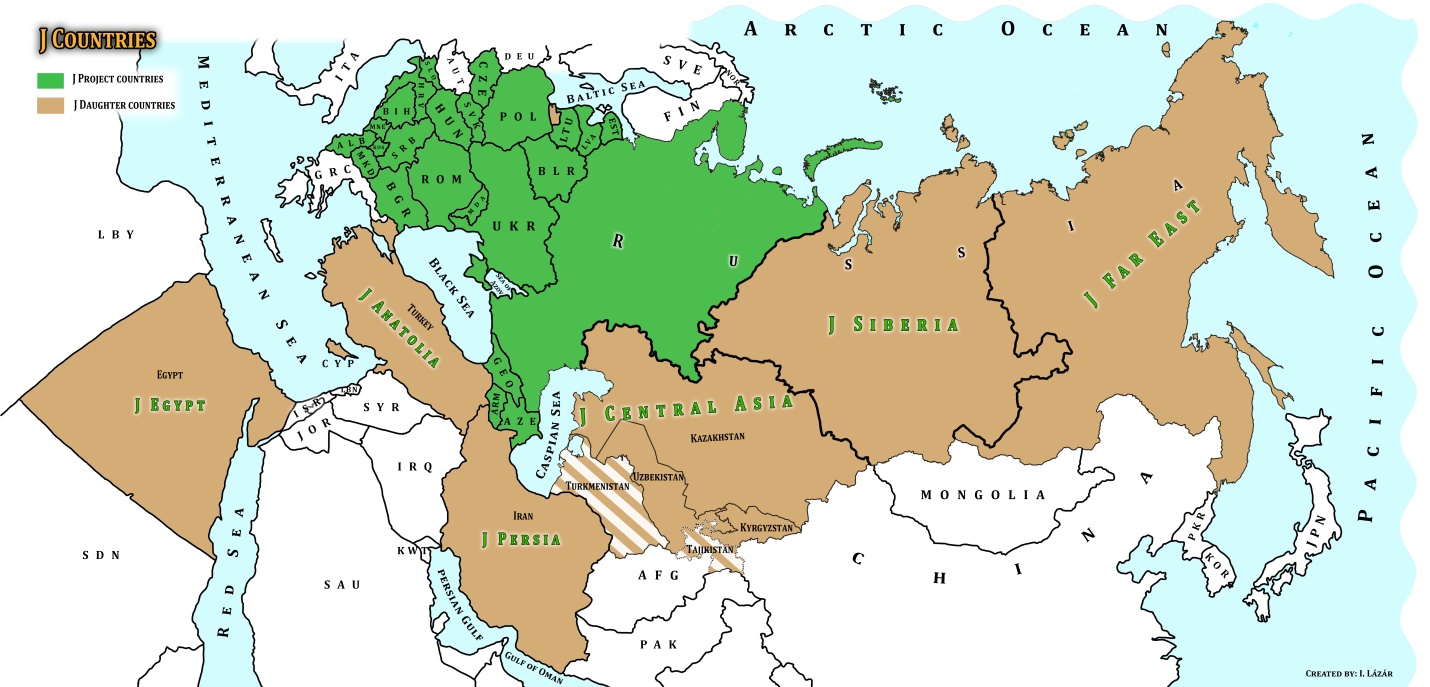

Supplement: Supplementary Figure 2 — Geographic extension of the J Project by 2020 which included 32 countries and 6 J Daughter (JD) Project regions chaired by N Rezaei (JD Persia, 2009), I Reisli (JD Anatolia, 2009), A Elmarshafi and N Galal (JD Egypt, 2009), I Tuzankina (JD Siberia, 2010), E Kovzel (JD Central Asia, 2012), and E Tcyvkina (JD Far East Russia, 2019). [file DataSheet_2.docx]

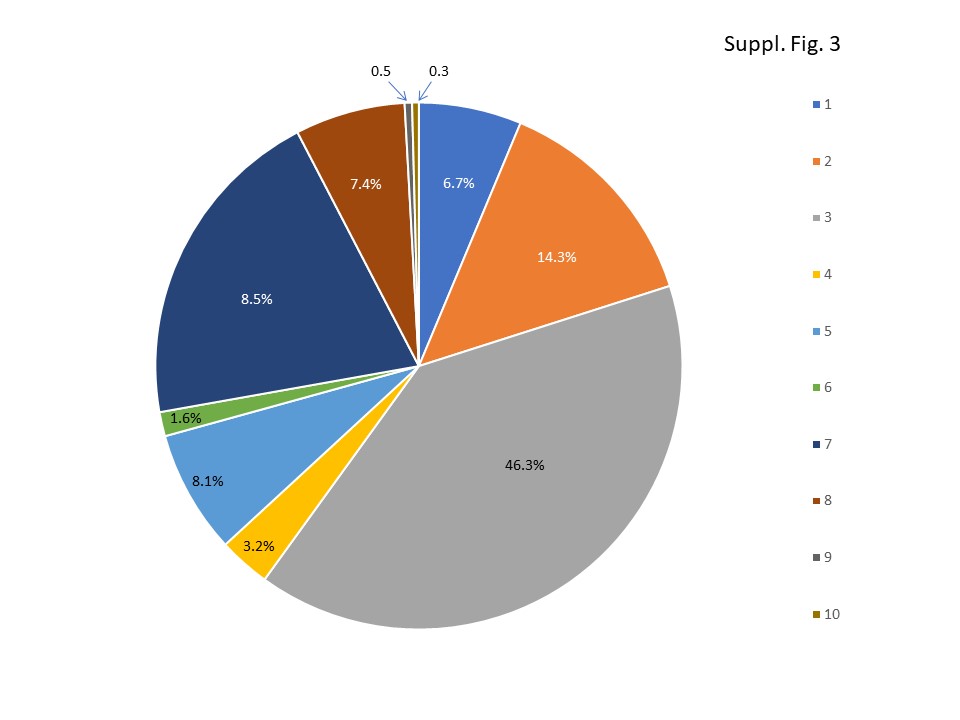

Supplement: Supplementary Figure 3 — Pie diagram showing the percentages of various inborn errors of immunity subgroups of patients reported to the J Project. [file Image_1.jpeg]

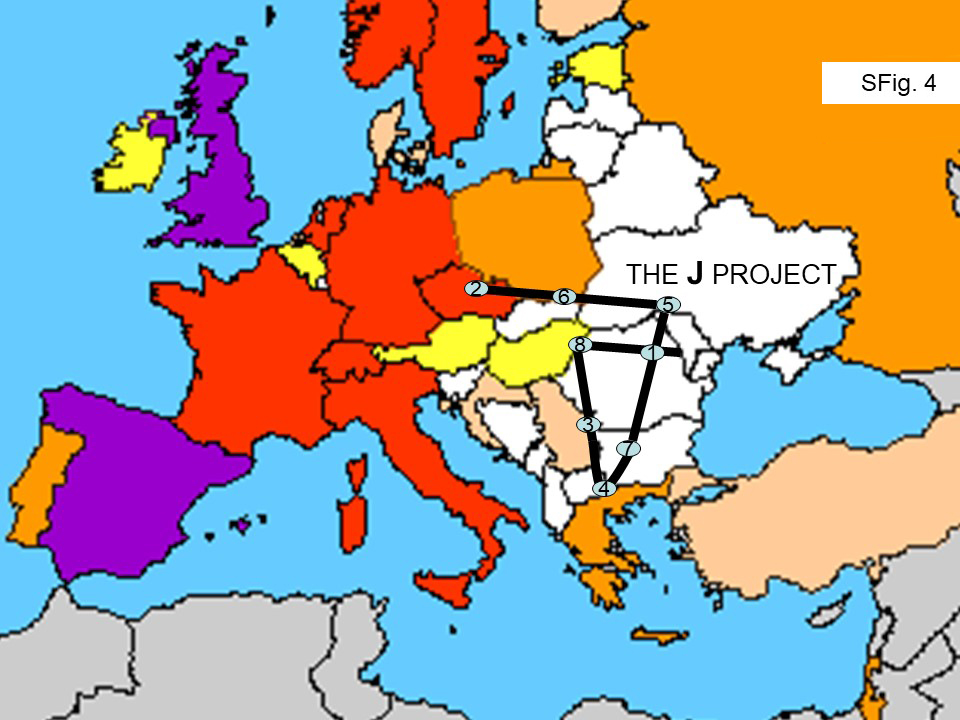

Supplement: Supplementary Figure 4 — The first 8 J Project meetings organized in Central Europe. [file Image_2.jpeg]
