# Supplementary material for: Care of patients with inborn errors of immunity in thirty J Project countries between 2004 and 2021
Source: Front Immunol. 2022 Dec 16;13:1032358. doi: 10.3389/fimmu.2022.1032358 (PMC9809467; doi:10.3389/fimmu.2022.1032358)
Supplement: Supplementary file 5 [file Table_1.docx]

**Suppl. Table 1. J Project Meetings 2004-2021**

| **Country** | | **SUM** | **2004** | **2005** | **2006** | **2007** | **2008** | **2009** | **2010** | **2011** | **2012** | **2013** | **2014** | **2015** | **2016** | **2017** | **2018** | **2019** | **2020** | **2021** |
| --- | --- | --- | --- | --- | --- | --- | --- | --- | --- | --- | --- | --- | --- | --- | --- | --- | --- | --- | --- | --- |
| 1 | Albania | **5** |  |  |  |  |  |  | 1 |  |  |  |  | 1 |  |  | 1 |  | 1 | 1 |
| 2 | Armenia | **4** |  |  |  |  |  |  |  |  |  |  |  |  |  |  | 1 | 1 | 1 | 1 |
| 3 | Azerbajan | **5** |  |  |  |  |  |  |  | 1 |  |  |  |  |  | 1 |  | 1 | 1 | 1 |
| 4 | Belarus | **8** |  |  | 1 |  |  | 1 |  |  | 1 |  |  | 1 |  |  | 1 | 1 | 1 | 1 |
| 5 | B&H | **1** |  |  |  |  | 1 |  |  |  |  |  |  |  |  |  |  |  |  |  |
| 6 | Bulgaria | **14** |  | 1 |  |  | 1 |  |  |  | 1 |  | 1 | 1 | 1 | 2 | 2 | 1 | 1 | 2 |
| 7 | Croatia | **1** |  |  |  |  |  |  | 1 |  |  |  |  |  |  |  |  |  |  |  |
| 8 | Czech Repub. | **14** | 1 | 1 | 1 |  |  |  |  |  | 1 | 2 | 1 | 1 | 1 | 1 | 1 |  | 1 | 2 |
| 9 | North Cyprus | **1** |  |  |  |  |  |  |  |  |  |  |  |  |  |  |  | 1 |  |  |
| 10 | Egypt | **3** |  |  |  |  |  | 1 | 1 |  |  |  | 1 |  |  |  |  |  |  |  |
| 11 | Estonia | **2** |  |  |  |  |  | 1 |  |  | 1 |  |  |  |  |  |  |  |  |  |
| 12 | Georgia | **4** |  |  |  |  |  |  |  |  |  |  |  |  |  |  | 1 | 1 | 1 | 1 |
| 13 | Hungary | **28** |  | 1 | 2 |  |  |  | 1 | 1 | 2 | 4 | 1 | 2 |  | 2 | 1 | 4 | 2 | 5 |
| 14 | Iran | **18** |  |  |  |  |  | 1 | 1 | 1 | 2 | 2 | 2 | 2 | 1 | 1 | 1 | 1 | 2 | 1 |
| 15 | Kazakhstan | **11** |  |  |  |  |  |  |  |  | 1 |  |  |  |  | 1 | 2 | 3 | 2 | 2 |
| 16 | Kyrgyzstan | **4** |  |  |  |  |  |  |  |  |  |  |  |  |  |  |  |  | 2 | 2 |
| 17 | Kosovo | **5** |  |  |  |  |  |  |  |  |  |  |  |  |  | 1 | 1 | 1 |  | 2 |
| 18 | Latvia | **5** |  |  |  |  | 1 |  |  | 1 |  |  |  |  |  |  |  |  | 1 | 2 |
| 19 | Lithuania | **6** |  |  |  |  |  |  |  |  |  |  |  |  | 1 | 1 | 1 | 1 |  | 2 |
| 20 | Poland | **18** | 1 | 1 |  |  |  | 1 |  | 1 | 2 | 1 | 1 |  | 1 | 1 | 1 | 1 | 4 | 2 |
| 21 | R.Macedonia | **8** | 1 |  |  | 1 |  |  |  | 1 |  |  |  |  |  |  | 1 | 1 | 2 | 1 |
| 22 | R.Moldova | **13** |  |  |  |  | 1 |  |  |  |  |  | 1 |  | 1 | 1 | 2 |  | 3 | 4 |
| 23 | Montenegro | **1** |  |  |  |  |  |  |  |  |  |  |  |  |  |  |  |  |  | 1 |
| 24 | Romania | **14** | 1 | 1 | 1 | 1 |  | 1 |  | 2 | 2 |  | 1 | 1 |  |  |  | 1 | 1 | 1 |
| 25 | Russia | **59** |  |  |  | 1 |  | 2 | 1 | 3 | 4 | 1 | 4 | 4 | 2 | 2 | 6 | 5 | 9 | 15 |
| 26 | Serbia | **2** | 1 |  |  |  |  |  | 1 |  |  |  |  |  |  |  |  |  |  |  |
| 27 | Slovakia | **10** |  |  |  |  |  |  |  | 1 | 1 | 1 | 1 | 1 | 1 | 1 | 1 | 1 |  | 1 |
| 28 | Slovenia | **12** |  |  |  | 1 |  | 1 |  |  | 1 | 2 | 1 | 1 | 1 | 1 | 1 |  | 1 | 1 |
| 29 | Tajikistan | **1** |  |  |  |  |  |  |  |  |  |  |  |  |  |  |  |  | 1 |  |
| 30 | Turkey | **30** |  |  |  |  |  | 1 |  | 1 | 3 | 1 | 3 | 2 | 3 | 3 | 3 | 4 | 2 | 4 |
| 31 | Ukraine | **25** | 1 | 1 |  | 1 | 1 | 1 | 1 | 1 | 1 | 1 | 1 | 1 | 1 | 1 | 1 | 2 | 4 | 5 |
| 32 | Uzbekistan | **10** |  |  |  |  |  |  |  |  |  |  |  |  |  |  | 2 | 4 | 1 | 3 |
| Edinburgh- ESID | | **1** |  |  |  |  |  |  |  |  |  |  |  |  |  | 1 |  |  |  |  |
| Lisbon- ESID | | **1** |  |  |  |  |  |  |  |  |  |  |  |  |  |  | 1 |  |  |  |
| **SUMMARY** | | **344** | **6** | **6** | **5** | **5** | **5** | **11** | **8** | **14** | **23** | **15** | **19** | **18** | **14** | **21** | **32** | **35** | **44** | **63** |
